# miR-21 attenuates lipopolysaccharide-induced lipid accumulation and inflammatory response: potential role in cerebrovascular disease

**DOI:** 10.1186/1476-511X-13-27

**Published:** 2014-02-07

**Authors:** Jun Feng, Antai Li, Jingyuan Deng, Yanhua Yang, Lili Dang, Yuanpeng Ye, Yuxin Li, Weiping Zhang

**Affiliations:** 1 Department of Cerebral vessels, First Affiliated Hospital of Medical College, Xi’an Jiaotong University, Xi’an 710061, China; 2 Department of Neurology, Xi'an Central Hospital, Xi'an 710003, China; 3 Department of Rehabilitation Medicine, First Affiliated Hospital of Medical College, Xi’an Jiaotong University, Xi’an 710061, China; 4 Department of Neurology, Shaanxi Armed Police Corps Hospital, Xi'an 710054, China; 5 Department of Neurology, Xingyuan Hospital, Yulin 719000, China; 6 Department of Neurology, The Second Affiliated Hospital, Xi’an medical college, Xi’an 710038, China

**Keywords:** miR-21, LPS, Atherosclerosis, Foam cells, Inflammation

## Abstract

**Background:**

Atherosclerosis constitutes the leading contributor to morbidity and mortality in cardiovascular and cerebrovascular diseases. Lipid deposition and inflammatory response are the crucial triggers for the development of atherosclerosis. Recently, microRNAs (miRNAs) have drawn more attention due to their prominent function on inflammatory process and lipid accumulation in cardiovascular and cerebrovascular disease. Here, we investigated the involvement of miR-21 in lipopolysaccharide (LPS)-induced lipid accumulation and inflammatory response in macrophages.

**Methods:**

After stimulation with the indicated times and doses of LPS, miR-21 mRNA levels were analyzed by Quantitative real-time PCR. Following transfection with miR-21 or anti-miR-21 inhibitor, lipid deposition and foam cell formation was detected by high-performance liquid chromatography (HPLC) and Oil-red O staining. Furthermore, the inflammatory cytokines interleukin 6 (IL-6) and interleukin 10 (IL-10) were evaluated by Enzyme-linked immunosorbent assay (ELISA) assay. The underlying molecular mechanism was also investigated.

**Results:**

In this study, LPS induced miR-21 expression in macrophages in a time- and dose-dependent manner. Further analysis confirmed that overexpression of miR-21 by transfection with miR-21 mimics notably attenuated lipid accumulation and lipid-laden foam cell formation in LPS-stimulated macrophages, which was reversely up-regulated when silencing miR-21 expression via anti-miR-21 inhibitor transfection, indicating a reverse regulator of miR-21 in LPS-induced foam cell formation. Further mechanism assays suggested that miR-21 regulated lipid accumulation by Toll-like receptor 4 (TLR4) and nuclear factor-κB (NF-κB) pathway as pretreatment with anti-TLR4 antibody or a specific inhibitor of NF-κB (PDTC) strikingly dampened miR-21 silence-induced lipid deposition. Additionally, overexpression of miR-21 significantly abrogated the inflammatory cytokines secretion of IL-6 and increased IL-10 levels, the corresponding changes were also observed when silencing miR-21 expression, which was impeded by preconditioning with TLR4 antibody or PDTC.

**Conclusions:**

Taken together, these results corroborated that miR-21 could negatively regulate LPS-induced lipid accumulation and inflammatory responses in macrophages by the TLR4-NF-κB pathway. Accordingly, our research will provide a prominent insight into how miR-21 reversely abrogates bacterial infection-induced pathological processes of atherosclerosis, indicating a promising therapeutic prospect for the prevention and treatment of atherosclerosis by miR-21 overexpression.

## Introduction

Atherosclerosis and its complications rank as the leading cause of death, representing nearly 29% of mortalities globally [1]. The large atherosclerotic plaque formation and subsequent rupture is the crucial mechanism underlying the onset of acute ischemic syndromes, including cerebral infarction, stroke, myocardial infarction, and sudden death [2-4]. It is commonly accepted that lipid-laden foam cell accumulation and inflammation in vessel walls are the hallmarks of the early stage of atherosclerosis, and then trigger a series of atherosclerotic complications [5].

Lipid deposition is the characteristic of atherosclerosis, and then forms the lipid core and earliest detected lesion, the fatty streak. It is known that the increasing macrophage foam cell formation induces the production of a large lipid-rich necrotic core, followed by the rupture of vulnerable plaque and subsequent thrombogenesis, a key trigger for acute cardiovascular diseases [6]. Blocking lipid deposition dramatically dampens atherosclerotic coronary lesions, indicating a potential target for atherosclerosis and cardiovascular events by the decrease of lipid levels [7,8].

Macrophages are believed to possess a pivotal function in lipid-laden foam cell formation and inflammation during atherosclerosis progression and plaque destabilization [9,10]. It is well known that macrophages can be activated by lipopolysaccharide (LPS) to uptake oxidized low-density lipoprotein (ox-LDL), which is a necessary step for macrophage foam cell production and the subsequent fatty streak formation. As a component of Gram-negative bacteria cell walls, LPS has been gradually demonstrated to be associated with cardiovascular disease [11-13]. When injection with endotoxin LPS in apolipo-protein E (apoE) deficient mice, the atherosclerotic lesion size is significantly increased [12,14]. Importantly, LPS can induce macrophage inflammation response and secrete abundant pro-inflammatory cytokines, which aggravate the atherosclerosis progress and lead to the instability of vulnerable plaques. Chronic administration of LPS in ApoE-/- mice obviously increases the production of inflammatory cytokines (such as TNF-α, IL-1β, IL-6, and MCP-1) and enhances the development of atherosclerosis [14]. Treatment with melittin dramatically recovers LPS-induced atherosclerotic lesions by the suppression of pro-inflammatory cytokines and adhesion molecules, suggesting an important anti-atherogenic strategy [15].

MicroRNAs (miRNAs) are known to be highly conserved, small non-coding RNA molecules (approximately 18–24 nucleotides), and represent a new class of gene regulators, which can interact with the 3’-untranslated region (3’-UTR) of a target gene to inversely regulate their target gene transcription or translation. Emerging evidences have demonstrated that miRNAs exert prominent roles in the inflammatory process and lipid accumulation in patients with coronary artery disease [16-18]. For example, miR-147 can act as a negative feedback regulator for Toll like receptor 4 (TLR4)-induced inflammatory responses [19]. Among these members, more researches have been focused on miR-21 as its significant roles in heart, tissue injury, inflammation, and cardiovascular diseases [20-22]. Recent research has confirmed a notable up-regulation of miR-21 in atherosclerotic plaques, indicating a pivotal effect on plaque destabilization [23]. However, the function of miR-21 in the progress of atherosclerosis and vulnerable plaques remains unknown.

In this study, we aimed to explore the effects of miR-21 on LPS-induced lipid accumulation and inflammation responses in macrophages. Furthermore, the underlying mechanism involved in this process was also discussed.

## Material and methods

### Reagents and antibodies

LPS (*Escherichia coli*, 055:B5) was obtained from Sigma (St. Louis, MO). Primary antibodies including antibodies against TLR4 and NF-κB p65 were purchased from Santa Cruz Biotechnology (Santa Cruz, CA). The corresponding horseradish peroxidase-conjugated secondary antibodies were from Calbiochem (La Jolla, CA). NF-κB inhibitor PDTC was from Sigma Chemical Co. (St. Louis, MO).

### Cell culture and treatment

Mouse RAW 264.7 monocyte/macrophage-like cell line was purchased from the American Type Culture Collection (ATCC, Manassas, VA). Cells were cultured at 37°C under 5% CO_2_ in DMEM supplemented with 10% FCS and 100 U/mL streptomycin-penicillin. Before stimulation with the indicated dose and times of LPS, cells were stimulated with anti-TLR4 antibody (10 μg/ml), or 30 μM NF-κB inhibitor PDTC for 4 h, prior to incubation with ox-LDL (50 μg/ml). Cells from the third to fifth passage were used in this experiment.

### Transfection

To specifically induce miR-21 expression in macrophages, the miRIDIAN™ miR-21 mimics was introduced. The miRIDIAN™ hairpin inhibitor was used to effectively silence the endogenous mature miR-21 function. The miR-2 mimics, scrambled control microRNA, anti-miR-210 inhibitor and anti-microRNA control inhibitor were obtained from Thermo Scientific (Lafayette, CO). For transfection, 0.4 nmol microRNA mimics or anti-microRNA inhibitors was mixed with 15 μl Geneporter 2 Transfection Reagent (GTS, San Diego), and were then transfected into 1 × 10^6^ cells for 6 h. After incubation with fresh medium for 48 h, cells were used for further experiments. miR-21 overexpression and inhibition were assessed using quantitative PCR.

### RNA extraction and quantitative real-time PCR

After stimulation with LPS, the total RNA from cells was isolated using the mirVana™ miRNA isolation kit according to the manufacturer’s instructions (Roche Diagnostics, Mannheim, Germany). To quantify the expression levels of miR-21 in cultured and transfected cells, TaqMan miRNA assay kits (Applied Biosystems, Foster City, CA) were introduced. Briefly, the obtained RNA was reverse-transcribed to synthesize the complementary DNA with the Oligo (dT) primer (Fermentas). Then, a sepcific primer for miR-21 and sno202 was obtained from Ambion to perform the TaqMan assays according to the manufacturers’ protocol. Additionally, sno202 was introduced as a normalizing control. The relative expression was calculated using 2^-^ΔΔ^CT^.

### Oil red O staining

After stimulation with LPS (100 ng/ml) and ox-LDL for 24 h, the cultured and transfected macrophages were washed with PBS three times, following fixation with 4% paraformaldehyde/PBS for 15 min. Then, cells were rinsed with ddH_2_O, and then the neutral lipids were stained using the freshly diluted 0.5% Oil red O solution (Sigma) for 10 min at 37°C. Cells were then rinsed with water, and hematoxylin was introduced to label the cell nuclei. The Oil red O-stained lipids in macrophage-derived foam cells were morphologically evaluated by microscopy.

### Lipid assay by high-performance liquid chromatography (HPLC)

The cellular lipids (total cholesterol, TC; cholesterol ester, CE) were analyzed as previously described [24]. Briefly, cells were rinsed with PBS for three times, and then lysed with 0.9% NaOH solution followed by homogenization in ice bath for 10 s. The BCA kit was used to evaluate the protein concentration, an equal volume of trichloroacetic acid was introduced and centrifuged for 10 min. Using stigmasterol to construct a standard curve first, and then the extraction procedure was repeated. Then, the samples were re-suspended in 100 μl of isopropanol-acetonitrile (v/v, 20:80) for 5 min. Ultimately, all the samples were placed on Agilent 1100 series HPLC (Wilmington, DE).

### Western blotting

Following rinses with PBS three times, the total protein extracts of RAW 246.7 cells were extracted using RIPA lysis buffer (Beyotime, Nantong, China), following the quantitative analysis of protein concentrations via the BCA assay (Pierce, Rockford, IL). For western blotting, the obtained protein was electrophoresed by SDS-polyacrylamide gel electrophoresis, and about 100 μg of protein was then transferred onto a polyvinylidene difluoride (PVDF) membrane in a semi-dry transblot apparatus. After incubation with buffer containing 5% nonfat dry milk in Tris-buffered saline with Tween (TBST) at 4°C overnight, the PVDF membrane was cultured with anti-TLR4 and anti- p65 NF-κB antibodies for 1 h at 37°C to probe the targeted protein. Following washed three times with TBST, HRP-conjugated secondary antibodies were added for 1 h. The LumiGLo reagent (KPL, Gaithersburg, MD) was used to visualize the bound antibodies. The protein expression levels were normalized by β-actin.

### Enzyme-linked immunosorbent assay (ELISA) assay

To analyze the levels of interleukin 6 (IL-6) and interleukin 10 (IL-10) in the transfected macrophages stimulated with LPS, the ELISA assay was introduced. Briefly, about 2 × 10^5^ cells were seeded into 24 well plates and incubated at 4°C overnight. The transfected cells were stimulated with LPS for 24 h, and the concentrations of IL-6 and IL-10 in supernatants were measured using ELISA DuoSet Development systems according to the manufacturer’s instructions (R&D Systems).

### Statistical analysis

All assays were performed in triplicate and numerical results were presented as mean ± SEM. SPSS 11.0 was used to analyze the data. The statistical significance of differences between groups was analyzed by Student *t*-test. A p value less than 0.05 was considered statistically significant.

## Results

### LPS induced miR-21 expression in macrophages

**Figure 1:**
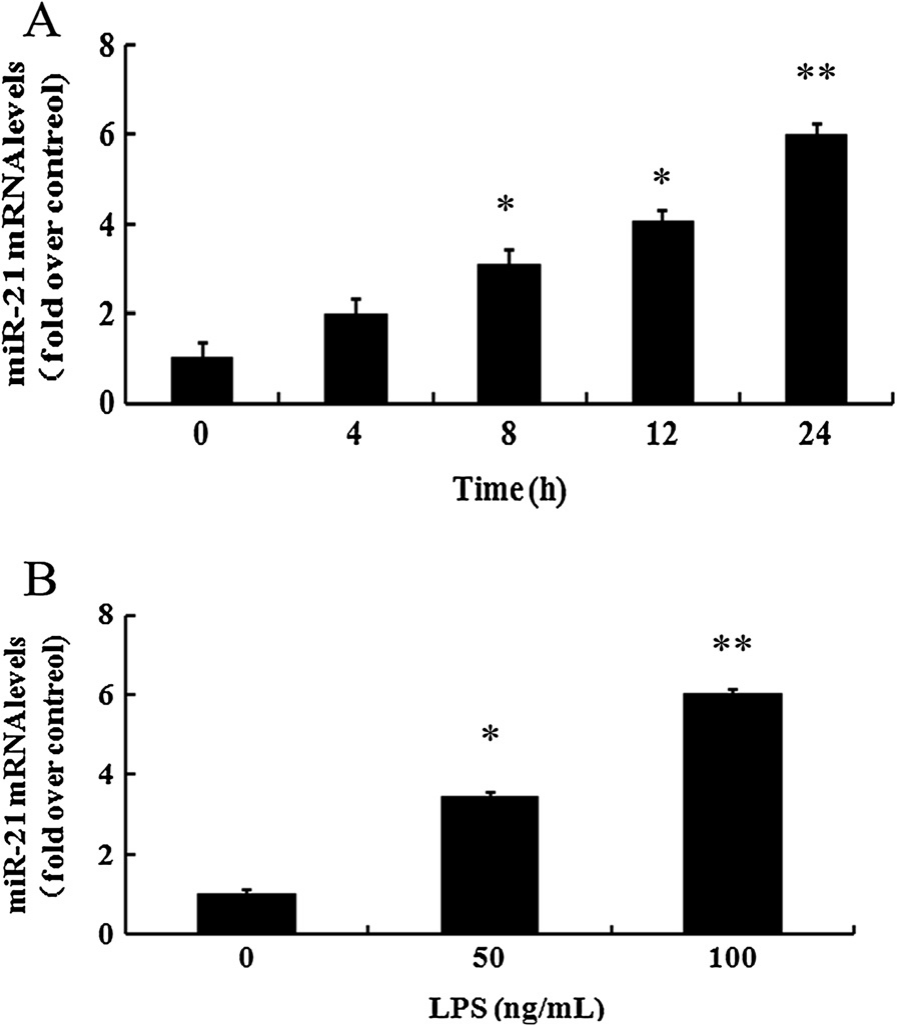
**LPS-induced expression of miR-21 in macrophages.** After stimulation with the indicated times **(A)** and doses **(B)** of LPS, the mRNA levels of miR-21 in macrophages were detected by quantitative RT-PCR. *P < 0.05 versus control group. **P < 0.01.

LPS is known to be critical for the progress of atherosclerotic plaques [13]. To assess the expression levels of miR-21 in LPS-induced macrophages, qRT-PCR was performed. As shown in Figure 1A, an obvious up-regulation of miR-21 mRNA levels was observed at 8 h post LPS stimulation. Simultaneously, treatment with inchmeal increased times of LPS stimulation, the mRNA levels of miR-21 were gradually up-regulated, indicating that LPS triggered a time-dependent increase in the expression levels of miR-21 mRNA in macrophages. After exposure to various doses of LPS (0, 50 and 100 ng/ml), the mRNA levels of miR-21 were about 3.4-fold and 6-fold over control at 50 ng/ml- and 100 ng/ml-treated groups, respectively (Figure 1B). Taken together, these results confirmed that LPS could trigger the expression of miR-21 in macrophages in a dose- and time-dependent manner.

### MiR-21 negatively regulated LPS-induced macrophage foam cell formation

**Figure 2:**
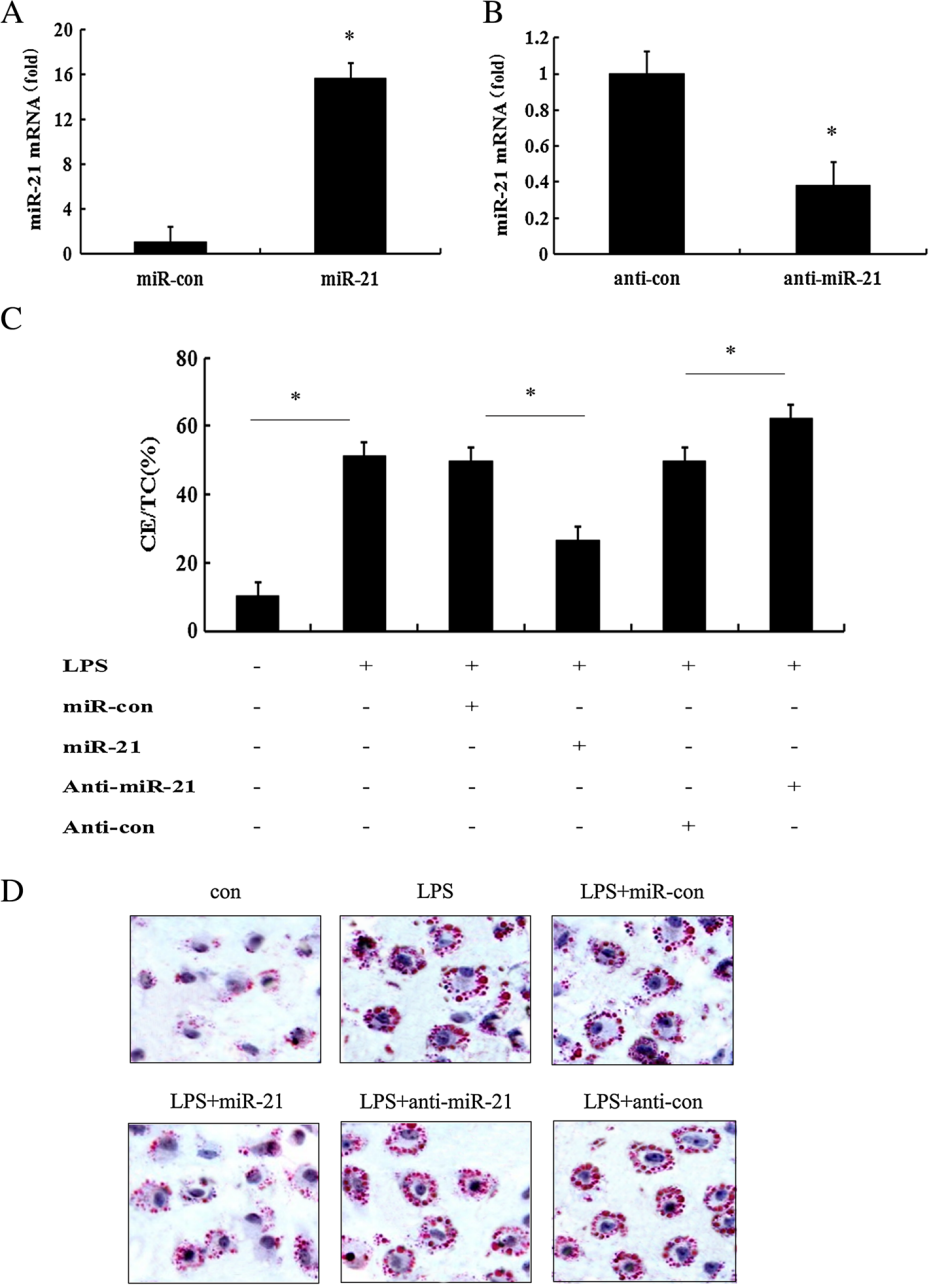
**Effect of miR-21 on lipid-laden foam cell formation in LPS-stimulated macrophages.** To investigate the function of miR-21 on LPS-induced lipid accumulation, cells were respectively transfected with miR-21 mimics, scrambled control microRNA, anti-miR-210 inhibitor and anti-microRNA control inhibitor for 6 h. **(A)** Overexpression of miR-21 in miR-21 transfected cells. *P < 0.05 versus control microRNA group. **(B)** Down-regulation of miR-21 in macrophages. *P < 0.05 versus anti-microRNA control inhibitor group. **(C-D)** After exposure to ox-LDL (μg/ml) for 24 h, the ratio of CE/TC and foam cell formation was assessed by HPLC assay **(C)** and Oil red O staining **(D)**. *P < 0.05.

Numerous reports have been confirmed that LPS can enhance the ability of macrophages to become foam cells, which is a pivotal trigger for atherosclerosis [14,25]. To investigate the function of miR-21 in LPS-induced foam cell formation, we overexpressed and silenced the expression levels of miR-21 in macrophages. After transfection with miR-21 mimics, a dramatic increase in miR-21 mRNA was observed in macrophages compared with the control group (Figure 2A). Moreover, anti-miR-21 inhibitor notably reduced the expression levels of miR-21 mRNA (Figure 2B). To further analyze the roles of miR-21 in LPS-induced lipid accumulation in macrophages, we performed the HPLC assay. As shown in Figure 2C, overexpression of miR-21 prominently attenuated LPS-induced lipid deposition, and the ratio of CE/TC decreased from 49.82% to 26.86%. Furthermore, silencing miR-21 mRNA levels through transfection with anti-miR-21 inhibitor, remarkably accelerated LPS-induced ratio of CE/TC. Additionally, Oil red O staining analysis suggested that overexpression of miR-21 obviously dampened LPS-triggered macrophage uptake of ox-LDL, and abrogated the formation of lipid droplets (Figure 2D). A corresponding increase in foam cell formation was also determined in anti-miR-21 inhibitors-transfected macrophages. Together, these results suggested that miR-21 overexpression inhibited LPS-induced foam cell formation, which was reversely enhanced when miR-21 mRNA levels were silenced in LPS-stimulated macrophages, indicating as a negative regulator of miR-21 in LPS-triggered macrophage foam cell formation.

### TLR4-NF-κB pathway was responsible for miR-21-mediated lipid deposition in LPS-stimulated macrophage

**Figure 3:**
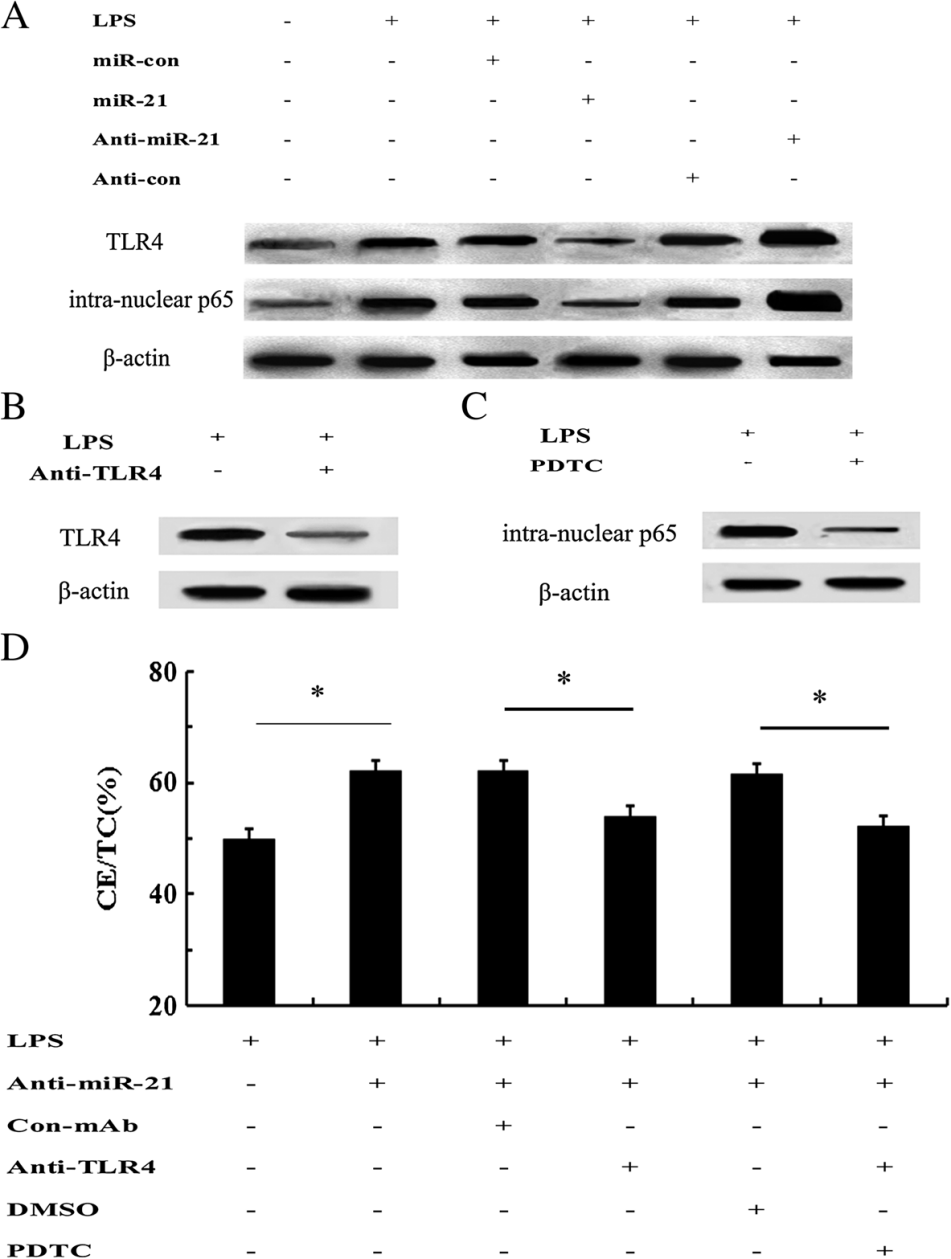
**miR-21 inhibited lipid accumulation by TLR4-NF-κB pathway. (A)** After overexpressed or silenced the expression levels of miR-21, the activation of TLR4 and intra-nuclear NF-κB p65 was demonstrated by western blotting analysis. **(B-C)** Before stimulation with 100 ng/ml LPS for 24 h, cells were stimulated with anti-TLR4 antibody (10 μg/ml), or 30 μM NF-κB inhibitor PDTC for 4 h. The silencing effect of TLR4 **(B)** and intra-nuclear NF-κB p65 **(C)** was evaluated. (**D**) The association between miR-21 and the TLR4-NF-κB pathway was analyzed by western blotting. *P < 0.05.

As a common receptor of LPS, TLR4 and its downstream signaling effector NF-κB are crucial for atherosclerotic plaque formation and coronary lesion progression [26,27]. To clarify the mechanism underlying miR-21-regulated lipid accumulation in macrophages, TLR4 and NF-κB pathway was introduced. Western blotting analysis ascertained that transfection with miR-21 mimics strikingly impeded the expression levels of TLR4, as well as intra-nuclear NF-κB p65 levels (Figure 3A). Consistently, down-regulation of miR-21 expression significantly increased the activation of TLR4 in LPS-induced macrophages, concomitant with the activation of intra-nuclear NF-κB p65. Together, our data indicated that miR-21 could dampen LPS-induced activation of TLR4-NF-κB pathway in macrophages.

To further assess the correlation between miR-21-regulated TLR4 signaling and lipid accumulation in LPS-induced macrophage, we silenced the TLR4-NF-κB pathway. As shown in Figure 3B, pretreatment with specific anti-TLR4 antibody dramatically abrogated TLR4 expression. Simultaneously, a significant inhibition of NF-κB activation was also manifested by preconditioning with the NF-κB inhibitor PDTC (Figure 3C). Further mechanism assays corroborated that anti-miR-21 inhibitor transfection significantly accelerated lipid deposition in LPS-stimulated macrophages, which was prominently attenuated by pretreatment with anti-TLR4 antibody, indicating that miR-21 silencing enhanced lipid accumulation by LPS-activated TLR4 pathway (Figure 3D). Moreover, a similar decrease in lipid accumulation was validated in PDTC-treated groups. Taken together, these results told that miR-21 majorly regulated lipid-laden macrophage foam cell formation stimulated with LPS via TLR4-NF-κB pathway.

### miR-21 mediated the production of inflammatory cytokines by TLR4-NF-kB in LPS-induced macrophages

**Figure 4:**
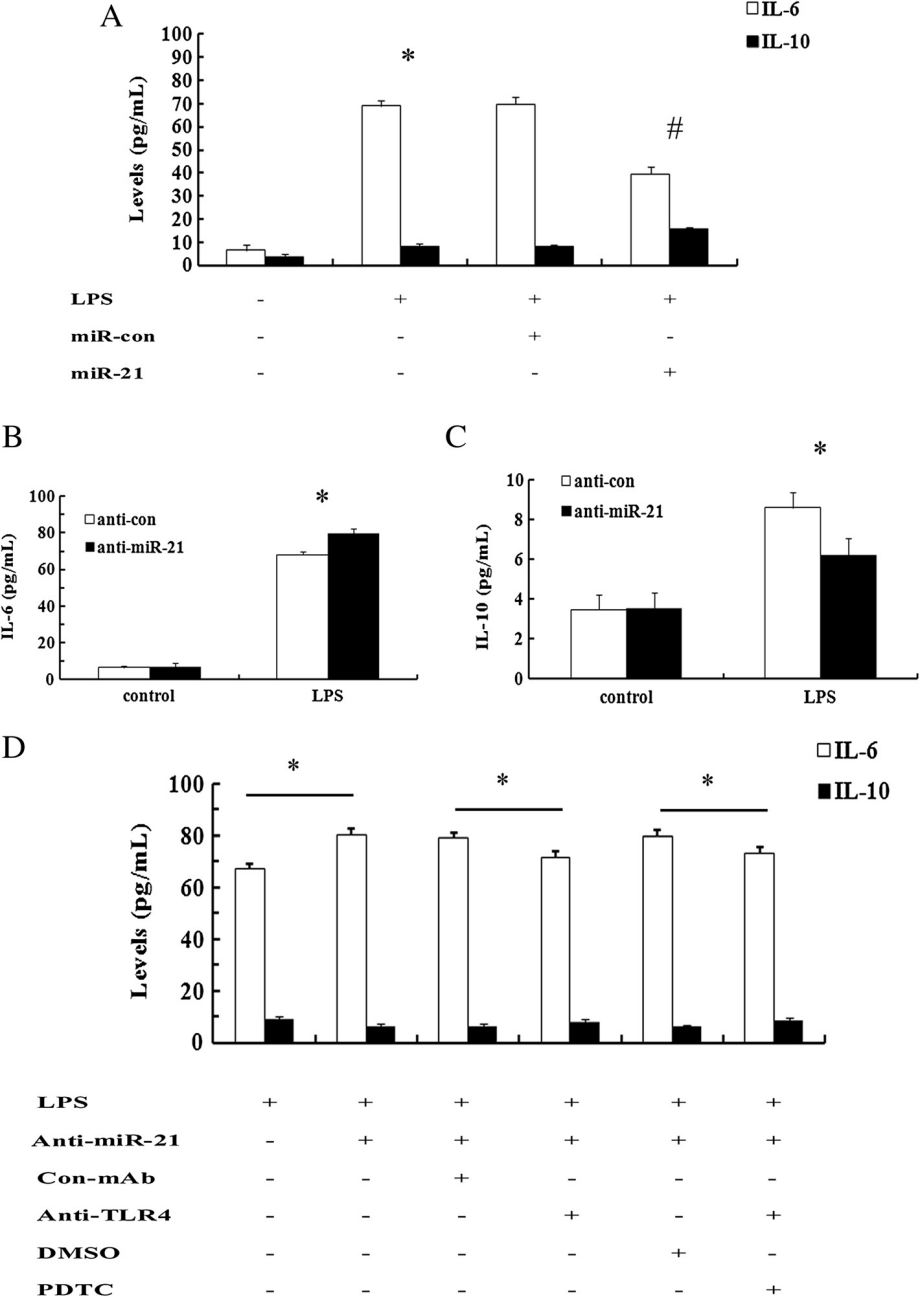
**miR-21 regulated the secretion of inflammatory cytokines in LPS-induced macrophages. (A)** Following transfection with miR-21, levels of IL-6 and IL-10 was detected by ELISA assay. *P < 0.05 versus LPS-untreated group. ^#^P < 0.05 versus LPS plus miR-con group. **(B-C)** The effect of miR-21 silencing on IL-6 and IL-10 levels in macrophages exposed to 100 ng/ml LPS. **(D)** Cells were pretreatment with anti-TLR4 antibody (10 μg/ml) or 30 μM NF-κB inhibitor PDTC for 4 h, and then LPS-induced IL-6 and IL-10 levels were detected in anti-miR-21 inhibitor-transfected cells. *P < 0.05.

During the progression of atherosclerotic plaques, the release of critical pro-inflammatory cytokines from macrophages such as IL-6, IL-12 and TNF-α, was considered to be pivotal [5,14]. To further assess the effects of miR-21 on LPS-induced inflammatory response in macrophages, we assessed the inflammation cytokine levels of IL-6 and IL-10 by ELISA assay. As shown in Figure 4A, LPS dramatically induced the production of IL-6 levels, and this increase was significantly decreased by miR-21 mimics transfection in macrophages. Furthermore, the levels of anti-inflammatory cytokine IL-10 was obviously enhanced compared to LPS-treated groups. Consistently, the inhibition of miR-21 expression with anti-miR-21 inhibitor transfection notably augmented IL-6 levels (Figure 4B), as well as an obvious decrease in IL-10 levels (Figure 4C). Therefore, all of these results showed that miR-21 down-regulated the pro-inflammatory cytokine IL-6 levels and up-regulated the anti-inflammatory cytokine IL-10 levels, indicating an important function on inflammatory response in macrophage-stimulated by LPS.

The activation of TLR-4 induces the production of inflammation cytokines to regulate the immune responses, which is a prominent contributor to atherosclerotic plaque formation and instability by the LPS/TLR4 signal transduction pathway [28,29]. To further clarify the underlying mechanism involved in miR-21-regulated macrophage inflammatory response, TLR-4 signaling was included. After silencing the activation of TLR-4 by specific antibody, the expression levels of IL-6 were strikingly attenuated compared to control in LPS-stimulated macrophages (Figure 4D). Moreover, blocking the activation of TLR-4 downstream effector NF-κB with PDTC, IL-6 levels was significantly impended in LPS and anti-miR-21-treated groups. For IL-10, its expression was correspondingly increased after preconditioning with TLR-4 specific antibody and NF-κB inhibitor PDTC, compared with LPS stimulation plus con-Amb and DMSO. Taken together, our data suggested that miR-21 could regulate inflammation response in macrophage stimulated by LPS via suppressing TLR-4-NF-κB signaling.

## Discussion

Cardiovascular disease has garnered increased interest and became the pre-eminent health problem worldwide as the leading cause of death and illness [1,3]. Atherosclerosis constitutes the single most crucial contributor to the outcome of cardiovascular diseases. Recently, numerous animal and cell experiments have focused on the miRNA profile in atherosclerotic processes, and an obvious up-regulation of miR-21 has been demonstrated in atherosclerotic plaques [23,30]. However, its function on the developmental progress of atherosclerotic plaques remains unclear. In this study, our results have manifested that miR-21 levels were significantly increased, and can negatively regulate lipid accumulation and inflammation cytokine secretion in LPS-stimulated macrophages by TLR-4-dependent signaling.

LPS, as one of the best studied immunostimulatory components of bacteria, has been proven to enhance lipid deposition and lipid-derived macrophage foam cell formation, as well as inflammatory cytokines release. All of these are the characterizations of atherosclerosis, and can regulate the pathological process of atherosclerosis and its complications. In this study, LPS dose- and time-dependently induced the expression of miR-21 mRNA. To clarify the roles of miR-21 in atherosclerosis, we analyzed the effect of miR-21 in LPS-induced lipid accumulation and inflammatory response in macrophages. After transfection with miR-21 mimics, the overexpression of miR-21 was induced in macrophages, and the corresponding down-regulation of it was also performed by anti-miR-21 inhibitor transfection. Lipid-laden foam cell formation is a critical trigger for the development of atherosclerosis. In this study, overexpression of miR-21 dramatically attenuated the ratio of CE/TC, indicating an obvious decrease in lipid accumulation in LPS-stimulated macrophages. Simultaneously, blocking of miR-21 expression accelerated LPS-induced lipid deposition in macrophages. Further analysis suggested that a notable reduction in foam cell formation was observed when overexpression of miR-21 in macrophages exposed to LPS. While in contrast to the control group, inhibiting miR-21 expression induced dramatically lipid droplets formation in macrophages stimulated with LPS. Together, our results suggested that miR-21 negatively regulated LPS-induced lipid accumulation in macrophages.

Toll-like receptors (TLRs) exerts multiple roles in atherosclerosis, and is highly expressed in atherosclerotic plaque. Among these members, TLR4 has drawn more attention during the development progress of atherosclerosis. TLR4 is known as the receptor of LPS, and its deficiency significantly attenuated aortic atherosclerosis in ApoE-/- mice [31]. Moreover, lipid accumulation in circulating monocytes was significantly reduced in TLR4-deficient mice. Growing evidence indicates that TLR4 plays a very important role in macrophage foam cells formation, indicating a critical roles of TLR4 in atherosclerosis via regulating lipid deposition [32,33]. To elucidate the underlying mechanism involved in miR-21-regulated lipid-laden macrophage foam cell formation, TLR4 pathway was discussed. As expected, miR-21 overexpression remarkably dampened the activation of TLR4 and its downstream NF-κB, while miR-21 expression inhibition reversely augmented the activation of TLR4-NF-κB. When blocking TLR4 expression with its specific antibody, LPS-induced lipid accumulation was strikingly decreased in macrophages transfected with anti-miR-21 inhibitor. Simultaneously, a similar reduction in lipid deposition was also confirmed when pretreatment with PDTC. Hence, these results suggested that miR-21 could negatively regulated lipid accumulation via TLR4-NF-κB pathway in LPS-stimulated macrophages, implying an important role in the development of atherosclerosis.

During the past decade, a prominent role for inflammation in atherosclerosis and its implications have been appreciated. Inflammation ranks as a major characterization for atherosclerosis, and the release of abundant inflammatory molecules will give rise to abnormal foam cell formation and initiate the development of atherosclerotic lesions. Blocking macrophage inflammation by TGR5 activation attenuates atherosclerosis lesions, indicating a potential therapeutic aspect in anti-atherosclerosis [34]. LPS is known as a potent inducer of the inflammatory response. Therefore, we further analyzed the effect of miR-21 in LPS-triggered inflammation in macrophages. Following transfection with miR-21, the levels of pro-inflammatory cytokine IL-6 was dramatically attenuated, accompany with an increase of anti-inflammatory cytokine IL-10. The corresponding changes of IL-6 and IL-10 were also confirmed when silencing miR-21 levels, indicating an important function of miR-21 on LPS-induced macrophage inflammation. As a key component of innate immune response, TLR4 possesses a pivotal role in the initiation and progression of atherosclerosis, and can regulate the inflammatory response in macrophages via its downstream NF-κB signaling [29,35]. To further elucidate the underlying mechanism involved in miR-21-mediated inflammation cytokines secretion, we blocked the activation of TLR4 and NF-κB. After blocking TLR4 expression, the increase in IL-6 and decrease in IL-10 was significantly mitigated in miR-21-silencing cells. The similar changes in IL-6 and IL-10 were also corroborated when preconditioning with PDTC in anti-miR-21 inhibitor-transfected macrophages. Together, these results told that miR-21 could regulate macrophage inflammation and lipid accumulation via the TLR4- NF-κB signaling pathway. However, the mechanism involved in miR-21-induced inhibitory effect on TLR4-NF-κB is still unclear, which needs to be explored in our next plan.

In conclusion, our research investigated for a potential role of miR-21 in atherosclerosis. In this study, LPS induced the expression of miR-21 in a time- and dose-dependent manner. Further analysis manifested that miR-21 negatively regulated lipid-laden foam cell formation and inflammatory responses in LPS-stimulated macrophages through the TLR4-NF-κB pathway, indicating a critical roles of miR-21 in the progression of atherosclerosis. Hence, the beneficial clinical effects of miR-21 overexpression in the prevention and treatment of atherosclerosis deserve further investigations.

## Abbreviations

miRNAs: microRNAs; LPS: Lipopolysaccharide; IL-6: Interleukin 6; IL-10: Interleukin 10; HLPC: high-performance liquid chromatography; ELISA: Enzyme-linked immunosorbent assay; TLR4: Toll-like receptor 4; NF-κB: Nuclear factor-κB; DMSO: Dimethyl sulfoxide; ox-LDL: oxidized low-density lipoprotein; TC: Total cholesterol; CE: Eholesterol ester.

## Competing interests

The authors have no financial conflicts of interest.

## Authors’ contributions

JF designed the study and prepare the manuscripts. ATL and JYD performed cell culture, treatment and lipid assay. YHY and LLD carried out the transfection and its detection of miR-21. YPY and YXL performed TLR-4- NF-κB and inflammation response assay. WPZ were involved in the statistical analysis. All authors read and approved the final manuscript.
